# Strong Association between Vitamin D Receptor Gene and Severe Acute Respiratory Syndrome coronavirus 2 Infectious Variants

**DOI:** 10.1055/s-0043-1761924

**Published:** 2023-02-16

**Authors:** Begimai Mamurova, Gokce Akan, Evren Mogol, Ayla Turgay, Gulten Tuncel, Emine Unal Evren, Hakan Evren, Kaya Suer, Tamer Sanlidag, Mahmut Cerkez Ergoren

**Affiliations:** 1 Department of Molecular Medicine, Institute of Graduate Studies, Near East University, Nicosia, Cyprus; 2 DESAM Research Institute, Near East University, Nicosia, Cyprus; 3 Laboratory of Medical Genetics, Near East University Hospital, Near East University, Nicosia, Cyprus; 4 Department of Medical Microbiology and Infectious Diseases, Faculty of Medicine, Kyrenia University; 5 Department of Medical Microbiology and Infectious Diseases, Faculty of Medicine, Near East University, Nicosia, Cyprus; 6 Department of Medical Genetics, Faculty of Medicine, Near East University, Nicosia, Cyprus

**Keywords:** COVID-19, *VDR*, polymorphism, *FokI*, *TaqI*

## Abstract

A coronavirus disease 2019 (COVID-19) disease, caused by the severe acute respiratory syndrome coronavirus 2 (SARS-CoV-2), has created significant concern since December 2019 worldwide. The virus is known to be highly transmissible. Heterogenic clinical features even vary more among SARS-CoV-2 variants from asymptomatic forms to severe symptoms. Previous studies revealed an association between COVID-19 and vitamin D deficiency resulting from its low levels in COVID-19 patients. To our knowledge, there is no scientific investigation that evaluates the direct association between SARS-CoV-2 variants of concern and vitamin D receptor ( *VDR* ) gene markers in Cyprus. Thus, the present study aimed to identify the putative impact of *VDR* gene polymorphisms on SARS-CoV-2 infection among different variants.

The nasopharyngeal swabs were taken from a total number of 600 patients who were admitted to Near East University Hospital COVID-19 Polymerase Chain Reaction (PCR) Diagnosis Laboratory for routine SARS-CoV-2 real-time quantitative reverse transcription PCR (RT-qPCR) test. The RT-qPCR negative resulting samples were taken as control samples ( *n* = 300). On the contrary, the case group consisted of patients who were SARS-CoV-2 RT-qPCR positive, infected with either SARS-CoV-2 Alpha ( *n* = 100), Delta ( *n* = 100), or Omicron ( *n* = 100) variants. Two *VDR* gene polymorphisms, *Taq* I-rs731236 T > C and *Fok* I-rs10735810 C > T, were genotyped by polymerase chain reaction-restriction fragment length polymorphism.

The mean age of the COVID-19 patient's ± standard deviation was 46.12 ± 12.36 and 45.25 ± 12.71 years old for the control group ( *p* > 0.05). The gender distribution of the patient group was 48.3% female and 51.7% male and for the control group 43% female and 57% male ( *p* > 0.05). Significant differences were observed in genotype frequencies of *FokI* and *TaqI* variants between SARS-CoV-2 patients compared to the control group ( *p* < 0.005). Furthermore, the risk alleles, *FokI* T allele and *TaqI* C, were found to be statistically significant (odds ratio [OR] = 1.80, 95% confidence interval [CI] = 1.42–2.29, OR = 1.62, 95% CI = 1.27–2.05, respectively) in COVID-19 patients. The highest number of patients with wild-type genotype was found in the control group, which is 52.9% compared with 17.5% in the case group. Moreover, most of the COVID-19 patients had heterozygous/homozygous genotypes, reaching 82.5%, while 47.1% of the control group patients had heterozygous/homozygous genotypes.

Our results suggested that patients with *FokI* and *TaqI* polymorphisms might tend to be more susceptible to getting infected with SARS-CoV-2. Overall, findings from this study provided evidence regarding vitamin D supplements recommendation in individuals with vitamin D deficiency/insufficiency in the peri- or post-COVID-19 pandemic.

A recent new coronavirus disease, the coronavirus disease 2019 (COVID-19) pandemic, has caused over 6.2 million deaths worldwide and still is a global health problem. 1 2 Viruses naturally adapt to environmental conditions over time. Therefore, viral variants might be raised due to mutations within the viral genome. These viral changes may allow the virus to spread more quickly and potentially trigger more serious complications. 3 The severe acute respiratory syndrome coronavirus 2 (SARS-CoV-2) genome has changed over time due to random mutations, its own recombination errors, and survival adaptation, resulting in the emergence of genetic variants that are believed to be highly infectious. 4

During the pandemic, the World Health Organization declared SARS-CoV-2 variants of concern (VoC), which included Alpha (B.1.1.7), Beta (B.1.351), Gamma (P.1), Delta (B.1.617.2), and Omicron (B.1.1.529). VoCs have a dramatically intensified attachment attraction in the receptor-binding domain (RBD)—human angiotensin-converting enzyme 2 complex due to mutations in the spike (S) protein RBD, enhancing the virus transmissibility. 5 Moreover, VoCs may modulate or even enhance their ability to replicate regardless of growing immunity in the population, which could be occurred *via* infection recovery or vaccines. 6 However, Alpha and Delta variants were presented to be more contagious and caused more deaths among the other viral genomic make-ups. 7

The interaction between human genomic variations and the COVID-19 disease severity still is unclear. 8 Early studies indicated an association between COVID-19 disease and vitamin D deficiency. 9 10

A steroid hormone, vitamin D, is a fat-soluble molecule that plays a vital role in bone strengthening, muscles, and the general well-being of individuals. The primary biological function of vitamin D is to keep average calcium and phosphorus blood levels. 11 Vitamin D also has anti-inflammatory functions, especially in viral infections, and can act as an immune modulator. 12 Moreover, multiple studies have found that vitamin D has a wide range of effects on cell growth, differentiation, cell death, immunomodulation, and genome stability. Recent research has also identified a correlation between vitamin D and cardiovascular disease, diabetes, cancer, autoimmune diseases, and infectious diseases, which means that vitamin D deficiency is associated with a broad range of diseases. 13

Limited sun exposure, advanced age, a sedentary lifestyle, and vitamin D receptor *(VDR)* gene polymorphisms might be the factors linked to a greater risk of vitamin D deficiency diseases. 14 However, a recent study showed that the young Turkish Cypriots population in Northern Cyprus was found to be vitamin D deficient, despite the fact that the Island of Cyprus receives constant sunlight for almost 9 months a year, and the Mediterranean diet is rich in vitamin D-containing foods. 14 15

Moreover, the polymorphisms on the *VDR* gene included the most well-known *TaqI* -rs731236 T > C, *ApaI* -rs7975232 A > C, *BsmI* -rs1544410 G > A, and *FokI* -rs10735810 C > T, which were associated with secretion of vitamin D and a higher risk of chronic diseases like autoimmune diseases, type 2 diabetes, and cancer. 16 17

Studies on the associations of COVID-19 prevalence and mortality rates and genetic variability of the hosts showed that *VDR* gene polymorphisms may play role in modulating COVID-19 infection and might be related to the survival of the patients and COVID-19 severity. 18 19 Therefore, *TaqI* and *FokI* gene polymorphisms might be contributed to modulating the response to vitamin D supplementation as linked to the improved reaction to supplementation as well as a predisposition to chronic diseases. Thus, this study aimed to investigate the possible impact of *VDR* gene markers on different SARS-CoV-2 infectious variants that caused long-pandemic.

## Materials and Methods

### Sample Collection

The nasopharyngeal swabs were taken from a total number of 600 patients who were admitted to Near East University Hospital COVID-19 Polymerase Chain Reaction (PCR) Diagnosis Laboratory for routine SARS-CoV-2 real-time quantitative reverse transcription PCR (RT-qPCR) test. RT-qPCR was performed for possible SARS-CoV-2 detection according to the manufacturer's guidelines (Uniplex RT-qPCR SARS-CoV-2 RT-qPCR Detection Kit, IKAS Medical, Nicosia, Northern Cyprus). The RT-qPCR negative resulting samples were taken as control samples ( *n* = 300). On the contrary, the case group consisted of patients who were SARS-CoV-2 RT-qPCR positive, infected with either SARS-CoV-2 Alpha ( *n* = 100), Delta ( *n* = 100), or Omicron ( *n* = 100) variants.

### Severe Acute Respiratory Syndrome Coronavirus 2 Variant Identification by Mutation Typing

The SARS-CoV-2 variant analysis was performed by the Multiplex SARS-CoV-2 VoC RT-qPCR Detection Kit (IKAS Medical, Nicosia, Northern Cyprus). The mutations including del69/70, N501Y, K417N, T478K, Y144del, and P681R were used to identify B.1.1.7 (Alpha), B.1.351 (Beta), P.1 (Gamma), B.1.617.2 (Delta), and B.1.1.529 (Omicron) variants. Delta variant was identified as follows: positive for 478K and P681R mutations and negative for the mutation H69-70 deletion, N501Y, K417N, and Y144del mutations. Omicron (BA.1) variant was identified as follows: positive for the H69-70 deletion, N501Y, T478K, K417N, and Y144del mutations, and negative for the P681R mutation. Lastly, the Alpha variant was identified as follows: positive for H69-70 deletion, N501Y, and Y144del mutations and negatives for T478K, K417N, and P681R mutations. All identified variants have been confirmed using whole-genome sequencing.

### Vitamin D Receptor Gene Polymorphisms Genotyping

The human genomic DNA was extracted from volunteered SARS-CoV-2 RT-qPCR positive and negative (control) cases using Uniplex RT-qPCR SARS-CoV-2 RT-qPCR Detection Kit (IKAS Medical, Nicosia, Northern Cyprus). Two *VDR* gene polymorphisms ( *Taq* I-rs731236 and *Fok* I-rs10735810) were genotyped by polymerase chain reaction–restriction fragment length polymorphism. Genotypes were determined according to the presence and absence of the two restriction sites that were analyzed, and alleles were designated with respect to actual base change according to the Ensembl ( http://www.ensembl.org/ ) genome browser and NCBI SNP database ( https://www.ncbi.nlm.nih.gov/SNP/ , dbSNP).

### Statistical Analysis

SPSS software (Statistical Package for the Social Sciences, SPSS Inc, Chicago, IL, United States, and version 25) was used to perform a statistical analysis of the data. Descriptive data and genotype data of the study group were demonstrated as mean ± standard deviation (SD) or number and frequency, where applicable. The Student's t-test and Mann–Whitney U test were used to make comparisons between normal and nonnormally distributed quantitative variables. Chi-square ( *x* ^2^ ) test was used to compare the genotype and allelic frequency distributions of *FokI* and *TaqI* polymorphisms between study groups. When the conditions for using the *x* 2 test were not accomplished, Pearson's chi-square test or Fisher's exact test were used to confirm the association of categorical variables between study groups. Hardy–Weinberg equilibrium was evaluated by Fischer's exact test. The odds ratio (OR) and 95% confidence interval (CI) were calculated using binary logistic regression analysis with codominant, dominant, additive, and recessive inheritance models. Akaike's information criterion was used to select the inheritance model. To assess group differences, the data were log-transformed to satisfy analysis of variance (ANOVA) criteria before being subjected to one-way ANOVA with Tukey's posthoc analysis. *TaqI* and *FokI* polymorphisms were assessed for their relative risks in SARS-CoV-2 variants infected individuals by estimating ORs and 95% CIs, which were considered separate outcomes. In all cases, differences were found important at *p* < 0.05.

## Results

In this study, we investigated the allelic frequencies and genotypic distribution between *VDR* gene *FokI* rs10735810 C > T and *TaqI* rs731236 T > C polymorphisms among patients infected by different SARS-CoV-2 VoC and compared with SARS-CoV-2 RT-qPCR negative subjects as a control group.

The study group included 300 COVID-19 patients who were infected by the SARS-CoV-2 Delta, Alpha, and Omicron BA.1 variants and 300 noninfected subjects as a control group. The mean age of the COVID-19 patient's ± SD was 46.12 ± 12.36 and 45.25 ± 12.71 years old for the control group ( *p* > 0.05). The gender distribution of the patient group was 48.3% female and 51.7% male and for the control group 43% female and 57% male ( *p* > 0.05).

### Allelic and Genotypic Distribution Frequency of Vitamin D Receptor *FokI* and *TaqI* Polymorphisms in the Studied Population

The genotypic distributions and allelic frequencies of *FokI* rs10735810 and *TaqI* rs731236 markers in COVID-19 patients and the control group are presented in Table 1 . Significant differences were observed in genotype frequencies of *FokI* and *TaqI* variants between SARS-CoV-2-infected patients and the control group ( *p* < 0.005) ( Table 1 ).

**Table 1:** The genotypic and allelic frequency distributions of Fok1 and Taq1 SNPs in studies groups

SNP	Genotypic frequencies *n* (%)		*p* -Value	Allelic frequencies			*X* 2	OR/CI (95%)	*p* -Value
Genotype	Cases ( *n* = 300)	Control ( *n* = 300)		Allele	Cases ( *n* = 300)	Control (n = 300)			
FokI-rs10735810									
TT	100(33.3)	160(53.3)							
TC	150(50)	120(36.7)	*0.001*	T/C	0.58/0.42	0.72/0.28	23.44	1.80/1.42–2.29	*0.001*
CC	50(16.7)	30(10)							
TaqI-rs731236									
TT	101(33.7)	150(50)							
TC	152(50.7)	120(40)	*0.001*	T/C	0.59/0.41	0.710/0.30	15.85	1.62/1.27–2.05	*0.001*
CC	47(15.7)	30(10)							

Abbreviations: CI, confidence interval; OR, odds ratio; SNP, single-nucleotide polymorphism.

Note: The study group included 300 COVID-19 patients who were infected by the SARS-CoV-2 Delta, Alpha, and Omicron BA.1 variant and 300 noninfected patients as a control group.

Note: The genotypic and allelic frequency distributions of polymorphisms between the groups were compared using x ^2^ and HWE tests. In all cases, differences were considered significant at *p* < 0.05.

Furthermore, the risk alleles, *FokI* T allele and *TaqI* C, were found to be statistically significant (OR = 1.80, 95% CI = 1.42–2.29, OR = 1.62, 95% CI = 1.27–2.05, respectively) in COVID-19 patients.

We also investigated genotypic distributions of *FokI* and *TaqI* polymorphisms together among the studied groups (case and control). The genotypes were grouped as follows: group 1: *FokI* TT (wild type) + *TaqI* TT (wild type) and group 2: *FokI* TC + CC (heterozygote + homozygote) + *TaqI* TC + CC (heterozygote + homozygote).

**Fig. 1:**
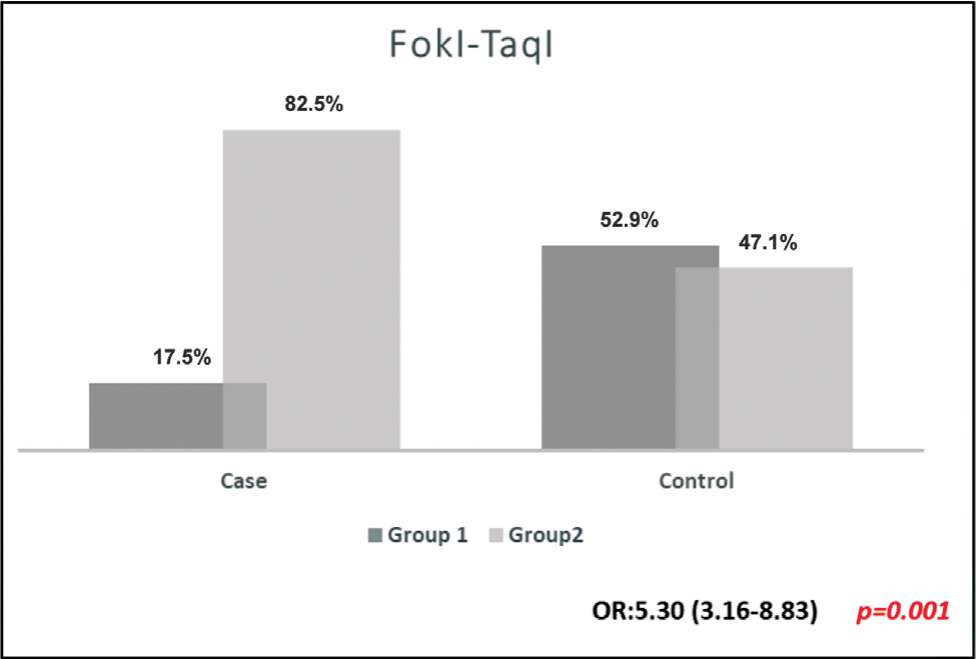
Comparison of *FokI* (rs10735810) and *TaqI* (rs731236) polymorphisms genotypes distributions among the study group. Group1: *FokI* TT (Wild Type) + *TaqI* TT (Wild Type) and Group2: *FokI* TC (Heterozygote+ Homozygote) + *TaqI* TC (Heterozygote+ Homozygote).

Thus, the highest number of patients with group 1 (wild-type genotype) was found in the control group, which is 52.9% compared with 17.5% in the case group. Moreover, most of the COVID-19 patients had group 2 (heterozygous/homozygous genotypes), reaching 82.5%, while 47.1% of the control group patients had group 2 ( Fig. 1 ).

These results suggested that patients with *FokI* and *TaqI* polymorphisms might tend to be more susceptible to getting infected with SARS-CoV-2.

### Vitamin D Receptor Gene Polymorphism Distributions among Severe Acute Respiratory Syndrome Coronavirus 2 Variants

**Fig. 2:**
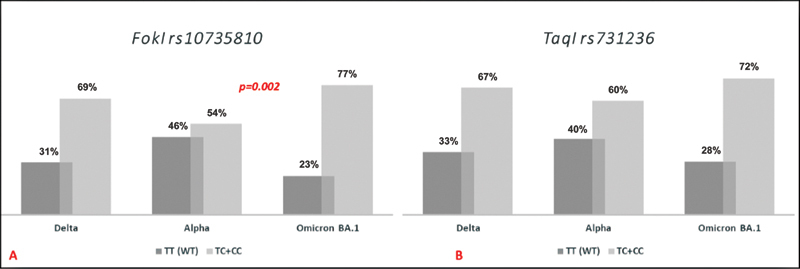
Genotype distributions among the SARS-CoV-2 variants. ( **A** ) *FokI* TT (wild type) and *FokI* TC + CC (heterozygote + homozygote). ( **B** ) TaqI TT (wild type) and TaqI TC + CC (heterozygote + homozygote).

Mutation analysis showed that *FokI* T allele was distributed in 69% of SARS- CoV-2 Delta variant-infected patients while 54% in SARS-CoV-2 Alpha variant infected patients. Additionally, 77% of SARS-CoV-2 Omicron BA.1 variant-infected patients carried at least one mutant allele for the same mutation ( Fig. 2A ). The differences in the distribution of the *FokI* polymorphism between the three variant groups were found as statistically significant ( *p* < 0.005).

Furthermore, *TaqI* T > C polymorphism was distributed in 67% of SARS-CoV-2 Delta variant subjects with at least one altered allele, while 60% of SARS-CoV-2 Alpha variant subjects carried at least one mutant allele. Also, 72% of the SARS-CoV-2 Omicron BA.1 variant infected patients carried at least one mutant allele ( Fig. 2B ). However, the genotype distribution for *TaqI rs731236* polymorphism among patients who were infected with different SARS-CoV-2 variants did not show any statistical significance.

### Analysis of Vitamin D Receptor Gene Polymorphisms Based on Genetic Inheritance Models

In genetic association studies, statistical power to detect disease susceptibility loci depended on the genetic models tested. Therefore, the genotype frequencies were further analyzed by four genetic models: additive, codominant, dominant, and recessive models ( Table 2 ). For *TaqI* , a significant association between this polymorphism and increased risk of COVID-19 patients were found in all four models, co-dominant genotype (TT) versus (CC) (OR = 2.66, 95% CI = 1.59–4.47, *p* = 0.001); codominant genotype (CC) versus (TT) (OR = 0.37, 95% CI = 0.25–0.72, *p* = 0.001); dominant (OR = 2.28, 95% CI = 1.64–3.18, *p* = 0.001); recessive (OR = 0.55, 95% CI = 0.34–0.90, *p* = 0.016); additive (OR = 0.56, 95% CI = 0.44–0.71, *p* = 0.001). Moreover, significant positive correlations between *TaqI* and risk of COVID-19 patients were also identified in codominant genotype (TT) versus (CC) (OR = 2.32, 95% CI = 1.38–3.92, *p* = 0.001); codominant genotype (CC) versus (TT) (OR = 0.43, 95% CI = 0.25–0.72, *p* = 0.001); dominant (OR = 1.97, 95% CI = 1.41–2.73, *p* = 0.005); recessive (OR = 0.60, 95% CI = 0.36–0.97, *p* = 0.03); additive (OR = 0.61, 95% CI = 0.48–0.78, *p* = 0.001).

**Table 2:** Analysis of *VDR* gene variations based on the four genetic inheritance models

SNP	Model of inheritance	OR (95% CI)	*p* -Value	AIC a
FokI-rs10735810	Codominant TT vs.CC CC vs.TT Dominant TT vs.TC + CC Recessive CC vs.TT + TC Additive TT vs.TC vs.CC	2.66 (1.59–4.47) 0.37 (0.25–0.72) 2.28 (1.64–3.18) 0.55 (0.34–0.90) 0.56 (0.44–0.71)	**0.001** **0.001** **0.001** **0.016** **0.001**	––16.19 30.66 22.68
TaqI-rs731236	Codominant TT vs. CC CC vs. TT Dominant TT vs. TC + CC Recessive CC vs. TT + TC Additive TT vs. TC vs. CC	2.32 (1.38–3.92) 0.43 (0.25–0.72) 1.97 (1.41–2.73) 0.60 (0.36–0.97) 0.61 (0.48–0.78)	**0.001** **0.001** **0.005** **0.03** **0.001**	––16.20 30.64 15.84

Abbreviations: AIC; Akaike's information criterion; CI, confidence interval; OR, odds ratio; SNP, single-nucleotide polymorphism.

Note: *p* -Values in bold remained significant after Bonferroni correction.

a The AIC: the preferred inheritance model is the one with the minimum AIC value.

b *p* -Value < 0.05 considered statistically significant.

## Discussion

COVID-19 disease created significant life concerns as a pandemic. The immune defenses of each patient were critical in limiting the risk of SARS-CoV-2 infection. COVID-19 pathophysiology might involve several signaling pathways and cellular components, including vitamin D2. 20 Vitamin D is an essential immune system modulator and has an anti-infective and immunomodulatory effect. 21 Recent studies indicated an association between COVID-19 disease and vitamin D deficiency, 9 10 also a study determined an interaction between pneumonia and low serum levels of 25-hydroxyvitamin D [25(OH) D]. 22 The primary end-point of the current study was to identify the putative interaction between *VDR* gene polymorphisms and SARS-CoV-2 infection among different VoCs.

In our study, we observed significant differences in genotype frequencies of *FokI* and *TaqI* variants between SARS-CoV-2-infected patients and the control group ( *p* < 0.005). Furthermore, the risk alleles, *FokI* T allele and *TaqI* C, were found to be statistically significant in COVID-19 patients. Also, when we grouped the studied SNPs according to genotypes ( *Group1: FokI TT* (wild type) *+ TaqI TT* (wild type), *Group2: FokI TC* *+* *CC* (heterozygote + homozygote) *+ TaqI TC* *+* *CC* (heterozygote + homozygote) and analyzed in our study population, the results showed that patients with *FokI* and *TaqI* polymorphisms might tend to be more susceptible to getting infected with the SARS-CoV-2. A previous study showed that the *VDR* gene *Fok1* TT genotype and/or T allele was associated with a greater risk of infection with enveloped viruses such as respiratory syncytial virus. 23 Moreover, a study in COVID-19 patients in the Cuban population showed evidence of an association between SARS-CoV-2 infection and the *VDR* gene *TaqI* polymorphism, 24 which also supported the findings of our study. A meta-analysis study indicated that vitamin D supplementation minimizes the chances of acute respiratory infections. 25 Another small-size study included 76 Spanish patients that were hospitalized due to COVID-19 disease and revealed that high doses of vitamin D application decreased the risk of intensive care unit admission. 26 Several studies demonstrated that taking vitamin D supplements could lower the possibility of viral infection severity as well as death. 25 Freitas et al implied that 76% of patients who died due to the COVID-19 were vitamin D deficient, whereas 59% of subjects had moderate symptoms and 64% of them had severe symptoms. 27 Another study, which included 77 older patients in France, found that taking vitamin D supplements on a regular basis for a year prior to a COVID-19 infection was related to a less severe condition and a higher chance of survival than if no vitamin D was taken. 28 These studies support the outcomes of the current study, demonstrating a strong association between vitamin D and COVID-19 disease.

However, none of the studies determined that *VDR* gene polymorphisms affect COVID-19 disease outcome as well as the SARS-CoV-2 variant impact on the disease and there was no scientific investigation that evaluates the association between SARS-CoV-2 VoC and *VDR* gene variations.

Furthermore, we investigated the putative impact of *VDR* gene polymorphisms on SARS-CoV-2 infection with different variants. The significant differences observed in the distribution of the *FokI* polymorphism between the three variant groups were found as statistically significant ( *p* < 0.005). We supposed that the *FokI* polymorphism may be related to the severity of COVID-19 in patients. Unfortunately, we could not access the patients' clinical data, which is one of the limitations of our study, and could not confirm the association between the *FokI* polymorphism and COVID-19 severity. However, findings of a recent study by Apaydin et al implied that serum 25 (OH) D levels had no relation to COVID-19 severity or mortality. Furthermore, it was discovered that *VDR* polymorphisms are independently related to the severity of COVID-19 and patient survival. 29 In addition, recent Israeli research based on the first two pandemic waves, which was prior to vaccine usage, discovered a risk of having severe COVID-19 in vitamin D-deficient individuals. 30

This case-control study is one of the few studies evaluating the association between SARS-CoV-2 VoC and *VDR* gene variations. Previous research had mostly used case reports and investigated mainly association between vitamin D levels and COVID-19 disease.

Nonetheless, due to certain limitations, our findings must be interpreted with caution. Limitations of this study included the lack of vitamin D serum level information for the participants and the lack of access to the patients' clinical data and not including another two important *VDR* gene polymorphisms *ApaI* -rs7975232 A > C and *BsmI* -rs1544410 G > A. Further clinical trials and research with a broader range of data are required to confirm the results of our study and further investigate the association between SARS-CoV-2 VoC and *VDR* gene variations.

## Conclusion

To conclude, the COVID-19 disease is still an important public health concern worldwide. This pandemic has severely harmed health care systems' ability to continue providing quality health care. As health care systems around the world struggle to meet the rising demand for COVID-19 care, sustaining preventive and therapeutic services is critical, especially for the most vulnerable populations such as children, the elderly, people with chronic illnesses, minorities, and people with disabilities.

It is very crucial to maintain preventive measures to avoid getting infected or minimize the severity of the disease by maintaining a safe distance from others and eating a well-balanced diet to be a healthier individual with a stronger immune system.

Our results displayed important differences in *VDR* polymorphisms' genotype distribution among different SARS-CoV-2 variants. The findings also implied that the patients with *FokI* and *TaqI* gene polymorphisms might be more susceptible to getting infected with SARS-CoV-2 VoC and provided evidence regarding vitamin D supplements recommendation in individuals with vitamin D deficiency/insufficiency in the peri- or post-COVID-19 pandemic.

## References

[1] Rauf A Abu-Izneid T Olatunde A COVID-19 pandemic: epidemiology, etiology, conventional and non-conventional therapies Int J Environ Res Public Health 2020 17 21 8155 33158234 10.3390/ijerph17218155 PMC7662254

[2] WHO Coronavirus (COVID-19) Dashboard, 2022. Accessed January 23, 01 2023 at:https://covid19.who.int/

[3] Raman R Patel K J Ranjan K COVID-19: unmasking emerging SARS-CoV-2 variants, vaccines and therapeutic strategies Biomolecules 2021 11 07 993 34356617 10.3390/biom11070993 PMC8301790

[4] Safari I Elahi E Evolution of the SARS-CoV-2 genome and emergence of variants of concern Arch Virol 2022 167 02 293 305 34846601 10.1007/s00705-021-05295-5 PMC8629736

[5] Shahhosseini N Babuadze G G Wong G Kobinger G P Mutation signatures and in silico docking of novel SARS-CoV-2 variants of concern Microorganisms 2021 9 05 926 33925854 10.3390/microorganisms9050926 PMC8146828

[6] Holick M F The vitamin D deficiency pandemic: approaches for diagnosis, treatment and prevention Rev Endocr Metab Disord 2017 18 02 153 165 28516265 10.1007/s11154-017-9424-1

[7] Gallagher J. (12 June 2021). “COVID: is there a limit to how much worse variants can get?”. BBC. Archived from the original on 15 June 2021. Retrieved 12 June 2021.

[8] Yi Y Lagniton P NP Ye S Li E Xu R H COVID-19: what has been learned and to be learned about the novel coronavirus disease Int J Biol Sci 2020 16 10 1753 1766 32226295 10.7150/ijbs.45134 PMC7098028

[9] Carpagnano G E Di Lecce V Quaranta V N Vitamin D deficiency as a predictor of poor prognosis in patients with acute respiratory failure due to COVID-19 J Endocrinol Invest 2021 44 04 765 771 32772324 10.1007/s40618-020-01370-x PMC7415009

[10] Weir E K Thenappan T Bhargava M Chen Y Does vitamin D deficiency increase the severity of COVID-19? Clin Med (Lond) 2020 20 04 e107 e108 32503801 10.7861/clinmed.2020-0301 PMC7385774

[11] Krawiec M Dominiak M The role of vitamin D in the human body with a special emphasis on dental issues: literature review Dent Med Probl 2018 55 04 419 424 30648367 10.17219/dmp/99051

[12] Charoenngam N Holick M F Immunologic effects of vitamin D on human health and disease Nutrients 2020 12 07 2097 32679784 10.3390/nu12072097 PMC7400911

[13] Wang H Chen W Li D Vitamin D and chronic diseases Aging Dis 2017 8 03 346 353 28580189 10.14336/AD.2016.1021 PMC5440113

[14] Kandemiş E Tuncel G Fahrioğlu U Temel ŞG Mocan G Ergören MÇ Natural selection at work? Vitamin D deficiency rates and rising health problems in young Turkish Cypriot professionals Cent Eur J Public Health 2021 29 02 130 133 34245553 10.21101/cejph.a6117

[15] Tuncel G Temel S G Ergoren M C Strong association between VDR FokI (rs2228570) gene variant and serum vitamin D levels in Turkish Cypriots Mol Biol Rep 2019 46 03 3349 3355 30977086 10.1007/s11033-019-04796-6

[16] Li L Wu B Liu J-Y Yang L-B Vitamin D receptor gene polymorphisms and type 2 diabetes: a meta-analysis Arch Med Res 2013 44 03 235 241 23506721 10.1016/j.arcmed.2013.02.002

[17] Lee Y H Bae S-C Choi S J Ji J D Song G G Associations between vitamin D receptor polymorphisms and susceptibility to rheumatoid arthritis and systemic lupus erythematosus: a meta-analysis Mol Biol Rep 2011 38 06 3643 3651 21110115 10.1007/s11033-010-0477-4

[18] Abdollahzadeh R Shushizadeh M H Barazandehrokh M Association of Vitamin D receptor gene polymorphisms and clinical/severe outcomes of COVID-19 patients Infect Genet Evol 2021 96 105098 34610433 10.1016/j.meegid.2021.105098 PMC8487094

[19] Al-Anouti F Mousa M Karras S N Associations between genetic variants in the vitamin D metabolism pathway and severity of COVID-19 among UAE residents Nutrients 2021 13 11 3680 34835935 10.3390/nu13113680 PMC8625365

[20] Raoult D Zumla A Locatelli F Ippolito G Kroemer G Coronavirus infections: Epidemiological, clinical and immunological features and hypotheses Cell Stress 2020 4 04 66 75 32292881 10.15698/cst2020.04.216 PMC7064018

[21] Aranow C Vitamin D and the immune system J Investig Med 2011 59 06 881 886 10.231/JIM.0b013e31821b8755 PMC3166406 21527855

[22] Mamani M Muceli N Ghasemi Basir H R Vasheghani M Poorolajal J Association between serum concentration of 25-hydroxyvitamin D and community-acquired pneumonia: a case-control study Int J Gen Med 2017 10 423 429 29180888 10.2147/IJGM.S149049 PMC5692194

[23] Laplana M Royo J L Fibla J Vitamin D Vitamin D Receptor polymorphisms and risk of enveloped virus infection: a meta-analysis Gene 2018 678 384 394 30092343 10.1016/j.gene.2018.08.017

[24] Peralta E M Rosales Y Z Mesa T C TaqI polymorphism of the VDR gene: aspects related to the clinical behavior of COVID-19 in Cuban patients Egypt J Med Hum Genet 2021 22 01 83 10.1186/s43042-021-00206-4 PMC8629595 38624931

[25] Martineau A R Jolliffe D A Greenberg L Vitamin D supplementation to prevent acute respiratory infections: individual participant data meta-analysis Health Technol Assess 2019 23 02 1 44 10.3310/hta23020 PMC6369419 30675873

[26] Entrenas Castillo M Entrenas Costa L M Vaquero Barrios J M “Effect of calcifediol treatment and best available therapy versus best available therapy on intensive care unit admission and mortality among patients hospitalized for COVID-19: A pilot randomized clinical study” J Steroid Biochem Mol Biol 2020 203 105751 32871238 10.1016/j.jsbmb.2020.105751 PMC7456194

[27] Freitas A T Calhau C Antunes G Vitamin D-related polymorphisms and vitamin D levels as risk biomarkers of COVID-19 disease severity Sci Rep 2021 11 01 20837 34675344 10.1038/s41598-021-99952-z PMC8531279

[28] Annweiler G Corvaisier M Gautier J Vitamin D supplementation associated to better survival in hospitalized frail elderly COVID-19 patients: the GERIA-COVID quasi-experimental study Nutrients 2020 12 11 3377 33147894 10.3390/nu12113377 PMC7693938

[29] Apaydin T Polat H Dincer Yazan C Effects of vitamin D receptor gene polymorphisms on the prognosis of COVID-19 Clin Endocrinol (Oxf) 2022 96 06 819 830 34919268 10.1111/cen.14664

[30] Dror A A Morozov N Daoud A Pre-infection 25-hydroxyvitamin D3 levels and association with severity of COVID-19 illness PLoS One 2022 17 02 e0263069 35113901 10.1371/journal.pone.0263069 PMC8812897

